# The Identification of Molecular Ploidy Status of Abnormal Pronuclear Zygotes Reveals a Significant Number of Euploid Blastocysts Available for Conception

**DOI:** 10.3390/biomedicines13010051

**Published:** 2024-12-28

**Authors:** Blair R. McCallie, Mary E. Haywood, Lauren N. Henry, Rachel M. Lee, William B. Schoolcraft, Mandy G. Katz-Jaffe

**Affiliations:** CCRM Genetics, 10290 Ridgegate Circle, Lone Tree, CO 80124, USAmandyk@ccrmivf.com (M.G.K.-J.)

**Keywords:** pronucleus, abnormal fertilization, infertility, pregnancy loss

## Abstract

**Objective:** Abnormally fertilized embryos are often discarded during in vitro fertilization due to the fact that known chromosomal ploidy abnormalities lead to implantation failure or pregnancy loss. The objective of this study was to determine if pronuclear numeration (PN) observed at fertilization check is representative of the true ploidy status of the subsequent developing blastocyst in order to maximize the number of viable embryos available for infertility patients and increase their chances of conception. **Methods:** Upon successful fertilization, pronuclear numeration was noted, and zygotes were cultured to the blastocyst stage. Biopsied trophectoderm cells were then lysed, and the isolated DNA was whole-genome amplified followed by library preparation. Next-generation sequencing was performed for PGT-A, and excess whole-genome amplified DNA was utilized for single nucleotide polymorphism beadchip array analysis. **Results:** At the time of fertilization check on day 1 of embryo development, when there were no visible pronuclei (*n* = 291), 56% of these 0PN blastocysts were confirmed to be diploid and normally fertilized. The remaining 41.9% were aneuploid, and 2.1% of the 0PN blastocysts contained only 23 haploid chromosomes. Upon analysis of the 1PN blastocysts (*n* = 217), just over a third (36.4%) only contained 23 haploid chromosomes (23XO), with another third (31.8%) identified as aneuploid, and surprisingly, the remaining third (31.8%) confirmed to be diploid and normally fertilized. In contrast, 50% of the 3PN blastocysts (*n* = 172) showed the presence of a third set of 23 parental chromosomes and were confirmed to be triploid (69XXY = 59.3% and 69XXX = 40.7%), with 41.9% identified as aneuploid and, interestingly, a small percentage (8.1%) confirmed to be diploid with normal fertilization. A very small proportion of biopsied blastocysts (0.63%) displaying the correct number of pronuclei for normal fertilization (2PN) were also identified as triploid with a third set of 23 parental chromosomes. To date, there have been 74 euploid embryo transfers from zygotes originally identified with an alternate pronuclear numeration, resulting in 16 ongoing pregnancies and 32 healthy live births, rates that match those typically observed with normally fertilized 2PN zygotes (>60%). **Conclusions:** A surprising number of blastocysts that were identified to have alternate pronuclear numeration at fertilization check on day 1 of embryo development were actually determined to be diploid with normal fertilization after molecular analysis. Accurate identification of haploid and tripoid zygotes is critical to prevent implantation failure and pregnancy loss and allows for the identification of all euploid embryos in a cohort, which has the potential to increase cumulative live birth rates for infertility patients.

## 1. Introduction

**Figure 1:**
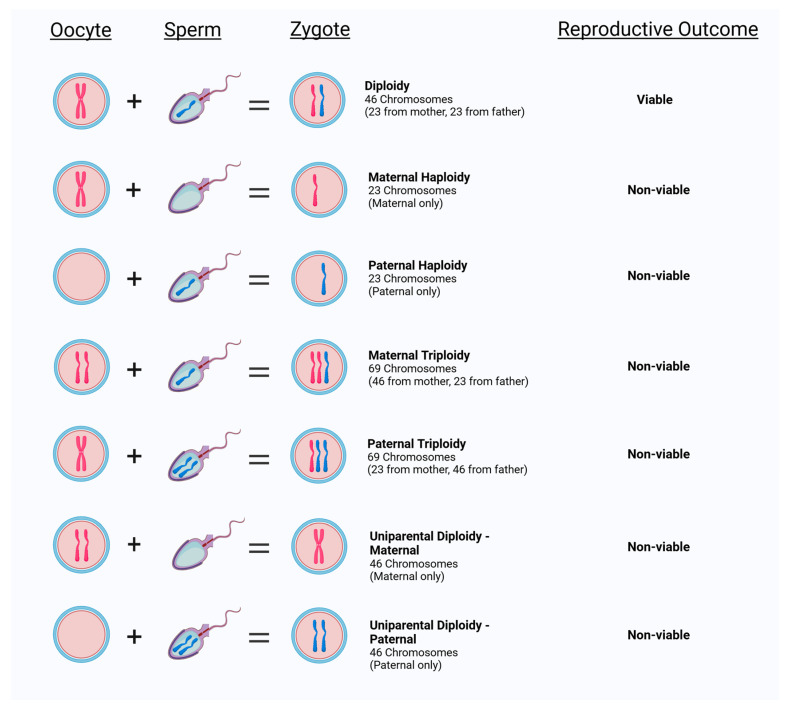
Types of ploidy conception observed in human reproduction.

During an in vitro fertilization (IVF) cycle, a fertilization check is performed 16–20 h after insemination where zygotes are morphologically assessed for the presence of pronuclei [1]. Normal fertilization requires proper segregation of the parental genomes for the developing embryo to acquire the correct biparental set of chromosomes [2]. Zygotes with two pronuclei (2PN) and two polar bodies are considered normally fertilized, with the assumption that each pronucleus originated from each of the two parental gametes (Figure 1) [3,4]. However, ploidy abnormalities are well documented in human conceptions. In fact, it is estimated that ~3–10% of inseminated oocytes fail to fertilize normally, and often when an alternate number of pronuclei are observed, these zygotes are discarded due to known chromosomal ploidy abnormalities that lead to implantation failure and/or pregnancy loss [5,6].

There are three different PN variations observed in IVF. First, the absence of any visible pronucleus (0PN), which could be the result of failed fertilization or that the pronuclei have disappeared by the time of fertilization check [7]. Second, the presence of only one pronucleus (1PN), which is typically considered to be a haploid embryo [8]. Haploid zygotes are not compatible with life due to the complete loss of half of the genome and have never been reported in prenatal testing [2]. The presence of only a single pronucleus can occur due to errors during fertilization, including failure of male or female chromatids to form a pronucleus, uncondensed sperm heads, ejection of the spermatozoon, fusion of the maternal and paternal pronucleus, or parthenogenesis (Figure 1) [9]. Lastly, three pronuclei (3PN), which are considered to be triploid embryos [5], are the most common abnormality associated with first-trimester pregnancy loss [10,11]. There are two types of triploidy: maternally inherited (digynic) or paternally inherited (diandric) (Figure 1). While paternally inherited triploidy commonly results in a molar pregnancy, maternally inherited triploidy results in intrauterine growth restriction, and both types almost always result in pregnancy loss [12]. The formation of 3PN zygotes during IVF after intracytoplasmic sperm injection (ICSI) is thought to occur as a result of a retention of the second polar body [13], but 3PNs can also arise via polyspermic fertilization or oocyte-derived meiotic failure [14]. Although rare, uniparental disomy (UPD) can also occur, where both sets of chromosomes are inherited from only one parent (Figure 1) [15].

Oocytes with an alternate number of pronuclei can develop into blastocysts that are morphologically indistinguishable from 2PN embryos, though at significantly lower rates [16]. 2PN zygotes are typically reported to have a blastocyst development rate of around 50%, whereas 1PN zygotes are between 26 and 32% [4,17,18]. 3PN zygotes also have reported lower blastulation rates compared to 2PN zygotes, at ~43% [9]. Additionally, while 3PN zygotes only have a prevalence of approximately 1–3% in all pregnancies, they are much more predominant in ICSI and IVF and account for 15–18% of cytogenetically abnormal cases among spontaneous abortions [13].

Studies have shown that some zygotes with an alternate number of pronuclei may actually have a normal chromosomal composition and could result in a euploid live birth [18,19,20]. However, it is also well documented that true abnormally fertilized embryos will result in spontaneous pregnancy loss, including triploid conceptions, like partial molar pregnancies, so it is imperative to confirm diploid fertilization prior to an embryo transfer [6,21,22]. Current next-generation sequencing (NGS) technologies utilized for preimplantation genetic testing for aneuploidy (PGT-A) include standardization where DNA is normalized to diploid and is, therefore, copy number neutral [8,15]. This renders the distinction between 23X and 46XX or 46XX and 69XXX impossible. However, single nucleotide polymorphism (SNP) analysis, in conjunction with NGS-based PGT-A, can be used to measure chromosome ploidy by generating data that can be evaluated using SNP B-allele frequencies for each chromosome [15,23,24,25,26]. To maximize the number of euploid embryos in an IVF cycle and minimize pregnancy failures/losses it is important to have the ability to identify every normally fertilized zygote. This study aims to determine if molecular identification of ploidy status can identify diploid fertilization in zygotes.

## 2. Materials and Methods

### 2.1. In Vitro Fertilization

All women underwent routine ovarian stimulation with an individualized physician-directed protocol that considered age, ovarian reserve, and prior responses. Conventional transvaginal ultrasound-guided oocyte aspiration was performed 35 h after gonadotropin-releasing hormone agonist and/or human chorionic gonadotropin triggering. Oocytes were then denuded of their cumulus cells and fertilized via standard intracytoplasmic sperm injection (ICSI) practices. Successful fertilization was assessed 16–18 h post-ICSI where PN numeration was noted and zygotes were sequentially cultured (Sage, CooperSurgical Fertility Solutions, Ballerup, Denmark) to the blastocyst stage. At the time of fertilization check on day 1 of embryo development, 680 patients undergoing IVF treatments between January 2021 and December 2023, who had zygotes with either no visible pronuclei (0PN; *n* = 291) or an alternate number of pronuclei (1PN; *n* = 217 or 3PN; *n* = 172), were included in this ethics-committee-approved study (protocol #20140458).

### 2.2. PGT-A

Alternate PN zygotes were individually tracked to the blastocyst stage, and upon identification of an inner cell mass (day 5, 6, or 7 of development), a trophectoderm biopsy was performed for PGT-A (Veriseq; Vitrolife Inc, Vastra Frolunda, Sweden). Briefly, herniating trophectoderm cells were aspirated into a biopsy pipette and detached from the blastocyst using an FDA-approved laser (Hamilton Thorne; Beverly, MA, USA) [27]. Biopsied blastocysts were then vitrified using the Cryotop method as previously described [28]. PGT-A analysis was completed by a single lab where whole genome amplification (WGA) was performed using the SurePlex DNA Amplification system (Vitrolife Inc, Vastra Frolunda, Sweden) according to the manufacturer’s protocol. Biopsied TE cells were lysed, and the genomic DNA was randomly fragmented and amplified. Following amplification, DNA quantity was determined using the High-Sensitivity (HS) Qubit Assay Kit (Life Technologies; Carlsbad, CA, USA). PGT-A was performed on TE biopsies using NGS (Veriseq PGS; Vitrolife Inc., Vastra Frolunda, Sweden). The NGS libraries were prepared from quantified WGA dsDNA using the VeriSeq™ PGS workflow (Vitrolife Inc, Vastra Frolunda, Sweden) followed by sequencing on a MiSeq instrument (Illumina Inc.; San Diego, CA, USA). In short, dsDNA was simultaneously fragmented and tagged with sequencing adapters by the VeriSeq™ PGS transposome, followed by a limited-cycle PCR amplification step, resulting in dual-indexed DNA. The library DNA was purified using AMPure XP beads (Beckman Coulter Life Sciences; Brea, CA, USA), removing the short library fragments. Pooled denatured libraries were sequenced on the MiSeq instrument (Illumina Inc.; San Diego, CA, USA) using single-end, dual index 36 base pair read sequencing. MiSeq Reporter software v2.5 (Illumina Inc.; San Diego, CA, USA) performed onboard secondary data analysis.

### 2.3. SNP Analysis

Aliquots of amplified DNA were used for whole-genome SNP analysis using the HumanKaryomap-12 Beadchip (Illumina Inc.; San Diego, CA, USA) in accordance with the manufacturer’s instructions. Briefly, samples were amplified and fragmented prior to an overnight hybridization to a beadchip containing ~300,000 informative markers. The next morning, beadchips were washed, and nucleotides were labeled to extend the hybridized primers. Following the staining of the primers, beadchips were scanned using the NextSeq 550 (Illumina Inc.; San Diego, CA, USA). Data files were processed using Illumina’s open-source BeadArrayFiles and gtc2vcf. For 0PN and 1PN blastocysts, GTC files were processed using Illumina’s open-source BeadArrayFiles v1.2.0 (https://github.com/Illumina/BeadArrayFiles, accessed on 5 May 2020) and the v1 manifest file. All samples underwent quality control checks to ensure allele dropout, call, and mis-call rates were within acceptable ranges based on the manufacturer’s guidelines.

### 2.4. Bioinformatic Data Analysis

**Figure 2:**
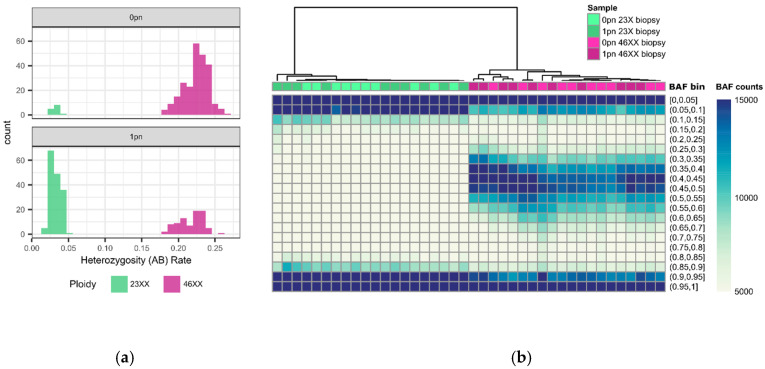
0pn and 1pn ploidy analysis: (**a**) histogram of heterozygosity (AB) rates for 0pn (*n* = 254) and 1pn (*n* = 242) biopsies. AB rates less than 0.05 are considered unfertilized (green), and rates greater than or equal to 0.18 are considered fertilized (pink). (**b**) Unsupervised hierarchical clustering heatmap of representative 0pn (*n* = 20) and 1pn (*n* = 20) biopsies that are fertilized (*n* = 20, pink) and unfertilized (*n* = 20, green). Biopsies determined by AB rate to be either 46XX or 23X cluster together, regardless of pronuclear scoring.

**Figure 3:**
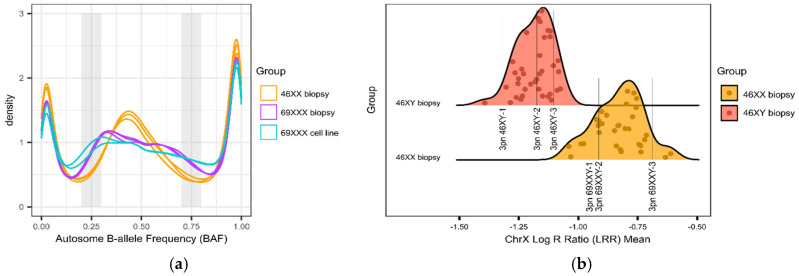
3pn ploidy analysis: (**a**) density plot of representative autosome B-allele frequency (BAF) for 46XX biopsies (*n* = 3, orange), 69XXX biopsies (*n* = 3, purple), and 69XXX cell lines (*n* = 2, blue). The shaded gray regions represent the two windows that are analyzed to determine ploidy status. (**b**) Density plot of ChrX log R ratio (LRR) means for representative euploid biopsies (*n* = 32 46XX, orange and *n* = 37 46XY, red). The two distributions are distinct and do not overlap. The LRR for representative examples of 3pn 46XY (*n* = 3) and 3pn 69XXY (*n* = 3) are shown.

The proportion of heterozygous genotypes was calculated and directly compared to the range of an established library of haploid, triploid, and diploid genome results. Samples with an overall heterozygosity (AB) rate ≤ 5% were considered haploid (based on the Illumina technical guide, where 5% is the mis-call rate that designates noise), while ≥18% were considered diploid (Figure 2). The diploid rate was calculated using historical in-house data from known euploid samples. For 0PN and 1PN blastocysts, GTC files were processed using Illumina’s open-source BeadArrayFiles v1.2.0 (https://github.com/Illumina/BeadArrayFiles, accessed on 5 May 2020) and the v1 manifest file. For 3PN blastocysts, GTC files were processed using the open-source gtc2vcf (https://github.com/freeseek/gtc2vcf, accessed on 5 May 2020) and the v1 manifest and cluster files. All 3PN zygotes were compared to a library of known euploid samples (*n* = 37 46XY and *n* = 32 46XX) (Figure 3). Samples exhibiting homozygosity on chromosome Y were tested for 69XXY, while all others were tested for 69XXX. Potential 69XXY samples were tested by assessing the log R ratio (LRR) of chromosome X and comparing that to the euploid database to determine whether one or two X chromosomes were present (Figure 3). A chromosome X mean LRR ≤ −1.0750221 (the maximum chrX mean LRR of a 46XY sample in the euploid database) is considered to be one copy, while a mean LRR ≥ −1.0396700 (the minimum mean chrX LRR of a 46XX sample in the euploid database) is considered to represent two copies. Potential 69XXX samples were tested by assessing the B-allele ratio (BAF) of autosomes. Two BAF regions (0.2–0.3 and 0.7–0.8) are compared to the euploid library. A BAF proportion < 5.89% within the 0.2–0.3 region and a BAF proportion < 4.31% within the 0.7–0.8 region were considered 46XX (Figure 3). Statistical analysis was performed using an unpaired *t*-test or ANOVA where appropriate.

## 3. Results

**Figure 4:**
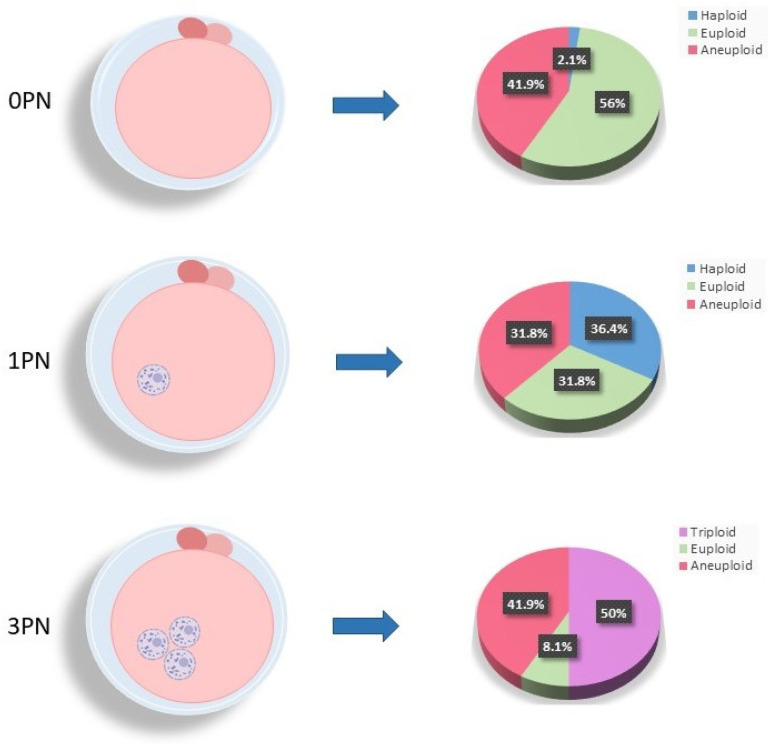
Molecular ploidy results for abnormal pronuclear zygotes.

At the time of fertilization check on day 1 of embryo development when no visible pronuclei (0PN) were observed (*n* = 291; average maternal age = 36. 0 ± 4.2; average paternal age = 37.3 ± 5.5), the vast majority of blastocysts (98%) were found to be normally fertilized, with 56% being euploid (*n* = 163; Figure 4) and 41.9% confirmed to be aneuploid (*n* = 122; Figure 4). Only a very small percentage (2.1%) of 0PN blastocysts were abnormally fertilized and haploid (*n* = 6; Figure 4). Upon analysis of the 1PN blastocysts (*n* = 217; average maternal age = 35.4 ± 4.0; average paternal age = 36.8 ± 5.8), just over a third (36.4%; *n* = 79; Figure 4) contained haploid chromosomes (23XO), with another third (31.8%; *n* = 69; Figure 4) identified as normally fertilized but aneuploid. The remaining third of the 1PN blastocysts (31.8%; *n* = 69; Figure 4) were confirmed to be normally fertilized and diploid. Half (50%) of all 3PN blastocysts showed the presence of a third set of parental chromosomes, confirming triploidy (*n* = 86; 69XXY = 59.3%; 69XXX = 40.7%; Figure 4). Additionally, 41.9% (*n* = 72; Figure 4) of the 3PN blastocysts were identified to be triploid and aneuploid, leaving only a very small percentage (*n* = 14; 8.1%; Figure 4) found to be diploid with normal fertilization. As expected, the average maternal age was significantly increased in chromosomally aneuploid embryos, regardless of PN numeration. Interestingly, a significant increase in mean maternal age was also observed for the triploid zygotes compared to 3pn zygotes with diploid fertilization (*p* < 0.05).

To date, there have been 74 euploid blastocyst transfers from zygotes originally identified with either 0PN (*n* = 53), 1PN (*n* = 17), or 3PN (*n* = 4), resulting in 16 ongoing clinical pregnancies and 32 healthy live births with no pregnancy or prenatal complications (Table 1). This included 29 IVF cycles where no other euploid embryos were available (8% of 0PN zygotes and 3% of 1PN zygotes). Four transfers led to a biochemical loss, two resulted in spontaneous miscarriage prior to the first ultrasound, one pregnancy was lost in the second trimester, and the remaining nineteen had a negative hCG (Table 1). The ongoing clinical pregnancy rate for 0PN embryo transfers appears to be trending higher than that of normally fertilized 1PN blastocysts (67.9% vs. 52.9%, ns, Table 1). However, the overall rate is similar to that of normally fertilized 2PN zygotes (64.8%). Three of the four normally fertilized 3PN embryos transferred thus far have resulted in healthy live births (Table 1).

## 4. Discussion

The transfer of abnormally fertilized embryos in an IVF setting is controversial due to the predicted resulting adverse outcomes. A triploid conception will result in spontaneous pregnancy loss, including partial molar pregnancy, whereas haploid embryos would typically fail to implant [6]. This study utilized SNP analysis to evaluate ploidy from a large cohort of embryos arising from 0PN, 1PN, and 3PN zygotes. A surprising number of blastocysts that were identified to have alternate pronuclear numeration at fertilization check on day 1 of embryonic development were actually found to be normally fertilized and containing two parental genomes.

Aside from aneuploidy, zygotes with no visible pronuclei were almost always diploid. It is evident that these embryos were most likely normally fertilized and two pronuclei were present, but they had disappeared prior to fertilization check. The utilization of a time-lapse system would enable the identification of pronuclei at any time during development. 1PN blastocysts, on the other hand, were diploid in roughly 2/3 of the cases. The other third, if transferred, would have resulted in pregnancy failure and/or loss. 3PN blastocysts were largely triploid, with only a small percentage showing a diploid chromosome constitution. However, performing additional SNP analysis prevented this small percentage of 3PN embryos from being discarded, which allows for a greater number of embryos available for transfer, leading to a higher probability of conception.

There are many suggested mechanisms that might lead to monopronucleation, including parthenogenesis, asynchronous pronuclei formation, early pronuclei fusion, and premature pronuclear breakdown, among others [3,29,30,31]. Apart from parthenogenetic activation, all other mechanisms have the possibility of producing normal diploid blastocysts [32]. Triploidy can be the result of a haploid oocyte fertilized by a haploid sperm, followed by a duplication of either the oocyte or sperm chromosomes. It can also occur when a haploid oocyte is fertilized by two haploid sperms or when an abnormal diploid oocyte is fertilized by a haploid sperm. A triploid conception will always result in spontaneous pregnancy loss, including a partial molar pregnancy, whereas haploid embryos would typically fail to implant [6].

Previous studies have shown the possibility of developmental perturbations, morphokinetic irregularities, and increased aneuploidy in abnormally fertilized zygotes [5,18,33]. Kratka et al. were the first group to utilize an SNP array platform to characterize ploidy in human blastocysts [15]. They found that triploidy was the most common abnormal fertilization outcome as a result of cell division errors largely occurring during meiosis. Another SNP array-based study looked at the developmental potential of abnormally fertilized embryos and found that 1PN-derived zygotes have considerably less blastocyst formation rates than that of 2PN, and abnormal chromosome constitution was more common in abnormally fertilized zygotes [34]. Tong et al. compared clinical outcomes from 0PN- and 1PN-derived blastocysts and found similar pregnancy, miscarriage, and live birth rates compared to those of their 2PN embryos [4]. Another study found similar pregnancy rates between 0PN, 1PN, and 2PN blastocysts, although they determined that 1PN zygotes resulted in higher miscarriage and lower live birth rates [21]. Our current study found similarly positive outcomes with an ongoing clinical pregnancy rate of 64.8% and low miscarriage rates with the transfer of a diploid, a euploid blastocyst. Girardi et al. performed a retrospective observational study of abnormal PN zygotes using a targeted-NGS approach and found similar rates of haploidy and diploidy in 0PN-, 1PN-, and 3PN-derived embryos as the current study [35]. Bradley et al. performed a retrospective analysis of 1PN embryos and found that they had lower developmental potential as determined by their reduced ability to blastulate by day 5. Additionally, they found that even though they were determined to have no genetic abnormalities via CGH analysis and biparental inheritance, they had lower-than-expected pregnancy outcomes [32].

There were 29 cycles in this study where no other euploid embryo was identified, offering these infertility patients an embryo available for transfer and a chance at conception. Of those 29 cycles, 16 thus far have undertaken a frozen embryo transfer, which has resulted in an ongoing pregnancy/live birth rate of 62.5%. This is the largest cohort, to our knowledge, that includes 0PN, 1PN, and 3PN embryos with clinical outcomes. A surprising number of zygotes with an alternate number of pronuclei at fertilization check were indeed diploid and can result in a live birth. Considering the fact that up to 10% of inseminated oocytes fail to normally fertilize in an IVF setting, this can amount to a significant proportion of embryos that may be discarded if there is no reliable way to determine true ploidy numeration and subsequent risk for pregnancy loss [36]. Adding SNP analysis for the identification of ploidy in cases of unknown pronuclei or alternate abnormal numbers of pronuclei allows for the identification of every diploid blastocyst during an IVF cycle. The most significant limiting factor to a successful reproductive outcome is the availability of a viable, euploid embryo for transfer. This current study shows promising clinical outcomes after the transfer of confirmed diploid blastocysts from an original zygote with an alternate number of pronuclei.

## Figures and Tables

**Table 1:** Confirmed diploid blastocyst frozen embryo transfer (FET) outcomes.

FET Outcome	Patients (All)	Percentage (of Total)	0PN	1PN	3PN
Negative βhCG	19	25.7%	14/53	4/17	1/4
Ongoing Pregnancy/Live Birth	48	64.9%	36/53	9/17	3/4
Positive βhCG	4	5.4%	2/53	2/17	0/4
Spontaneous Miscarriage	3	4.0%	1/53	2/17	0/4

## Data Availability

Data are contained within the article.
